# Cell migration leads to spatially distinct but clonally related airway cancer precursors

**DOI:** 10.1136/thoraxjnl-2013-204198

**Published:** 2014-02-18

**Authors:** Christodoulos P Pipinikas, Theodoros S Kiropoulos, Vitor H Teixeira, James M Brown, Aikaterini Varanou, Mary Falzon, Arrigo Capitanio, Steven E Bottoms, Bernadette Carroll, Neal Navani, Frank McCaughan, Jeremy P George, Adam Giangreco, Nicholas A Wright, Stuart A C McDonald, Trevor A Graham, Sam M Janes

**Affiliations:** 1 Lungs for Living Research Centre, UCL Respiratory, University College London, London, UK; 2 Department of Respiratory Medicine, University of Thessaly School of Medicine, Larissa, Greece; 3 Department of Pathology, University College London, London, UK; 4 Department of Thoracic Medicine, University College London Hospital, London, UK; 5 Department of Biochemistry, University of Cambridge, Cambridge, UK; 6 Department of Asthma, Allergy and Respiratory Science, King's College London, London, UK; 7 Histopathology Laboratory, Cancer Research UK London Research Institute, London, UK; 8 Centre for Digestive Diseases, Barts and the London School of Medicine and Dentistry, Queen Mary University of London, London, UK; 9 Centre for Evolution and Cancer, UCSF Helen Diller Family Comprehensive Cancer Center, San Francisco, California, USA

**Keywords:** Lung Cancer, Airway Epithelium

## Abstract

**Background:**

Squamous cell carcinoma of the lung is a common cancer with 95% mortality at 5 years. These cancers arise from preinvasive lesions, which have a natural history of development progressing through increasing severity of dysplasia to carcinoma in situ (CIS), and in some cases, ending in transformation to invasive carcinoma. Synchronous preinvasive lesions identified at autopsy have been previously shown to be clonally related.

**Methods:**

Using autofluorescence bronchoscopy that allows visual observation of preinvasive lesions within the upper airways, together with molecular profiling of biopsies using gene sequencing and loss-of-heterozygosity analysis from both preinvasive lesions and from intervening normal tissue, we have monitored individual lesions longitudinally and documented their visual, histological and molecular relationship.

**Results:**

We demonstrate that rather than forming a contiguous field of abnormal tissue, clonal CIS lesions can develop at multiple anatomically discrete sites over time. Further, we demonstrate that patients with CIS in the trachea have invariably had previous lesions that have migrated proximally, and in one case, into the other lung over a period of 12 years.

**Conclusions:**

Molecular information from these unique biopsies provides for the first time evidence that field cancerisation of the upper airways can occur through cell migration rather than via local contiguous cellular expansion as previously thought. Our findings urge a clinical strategy of ablating high-grade premalignant airway lesions with subsequent attentive surveillance for recurrence in the bronchial tree.

**Key messages**

**What is the key question?**

- Are spatially distinct precancerous lesions of the airways related?

**What is the bottom line?**

- We demonstrate that cells from carcinoma in situ lesions are capable of migrating across histologically normal epithelium and establishing new clonal lesions and hence propose a novel mechanism of ‘field cancerisation’ within human airways with important implications for lung cancer clinicians.

**Why read on?**

- Using longitudinal tracking of lesions, our study provides a novel answer to arguments that have run since the publication of the Franklin paper (1997), which noted an identical mutation in separate premalignant lesions leading to speculation that either the mutation occurred across the whole bronchial tree or that identical mutations were occurring independently generating new clones.

## Introduction

The proposed progression model for squamous cell carcinoma (SqCC) of the lung is a stepwise change in morphology from the early, non-specific change of squamous metaplasia to mild, moderate and severe dysplasia (SD) to carcinoma in situ (CIS) and finally invasive cancer.^1^ Although preinvasive lesions such as SD and CIS are thought to be precursors of invasive SqCC, not all progress to malignant tumours.^1^ We have previously documented that a minority of high-grade lesions progress to invasive carcinoma within 2 years of follow-up with the majority remaining static or regressing.^2^ Importantly, the presence of these preinvasive lesions predisposes to cancer development at remote sites.^2^ ^3^

An increasing body of evidence suggests that genetic mutations accumulate at a preneoplastic stage, long before clinical or histological detection.^4^ Clonal expansion of these mutants may not cause morphological change but can predispose the epithelium to subsequent tumour development,^5^ a process known as ‘field cancerisation’. Field cancerisation demonstrated in several organs^6–12^ was first proposed by Slaughter *et al*.^13^ They observed oral cancers to grow multifocally at sites surrounded by areas of abnormal, precancerous cells and proposed the regional preconditioning of epithelium by an unknown carcinogenic insult.^13^ Braakhuis *et al*^14^ subsequently suggested the precancerous field was monoclonal in origin, a definition that fits well with the observation of several preinvasive lesions carrying the same rare somatic *TP53* mutation in the bronchial tree of a patient at autopsy.^15^

Previous studies investigating the accumulation of somatic changes in preinvasive and invasive lesions used specimens taken at a single time point, often from surgical resection specimens.^16–18^ The development of autofluorescence bronchoscopy (AFB) has improved detection of preinvasive lesions in the lung,^19^ providing the means for the longitudinal tracing and facilitating the anatomical and biological study of the natural history of preinvasive lesions in situ. We developed our longitudinal study of preinvasive lesions to help delineate both their clonal and temporal relationship.

In our study, we wished to answer whether these lesions occur in a field of clonally related epithelium, whether lesions with identical mutations occur independently or whether they occur after migration through a genetically unrelated epithelium and then expand in a new favourable environment or ‘niche’ at a distant location. We combine temporal mapping of preinvasive lung lesions using AFB with mutation analysis of biopsy samples to measure the clonal expansion of these lesions within the tracheobronchial tree and delineate the extent and mechanism of ‘field cancerisation’. We focus on individuals with rare tracheal CIS disease and examine their clonal and temporal relationship with other preinvasive lesions. The first patient described gives a unique insight into the mechanism of preinvasive epithelial cell migration and subsequent clonal expansion over a 12-year period, the additional four patients further demonstrate that the occurrence of tracheal CIS is usually preceded by clonally related but spatially distinct lesions more distal in the airway.

## Methods

### Patient recruitment

The University College London Hospitals (UCLH) Early Lung Cancer Surveillance Program uses AFB to assess patients at high risk for lung cancer. Eligibility criteria for inclusion into the programme involve the detection of one or more preinvasive lesions in the absence of clinically or radiologically detected invasive carcinoma or development of preinvasive lesions at remote sites from the site receiving curative treatment for carcinoma. All patients in the programme are investigated with repeated AFB and CT or positron emission tomography/CT . Full details of the surveillance protocol have been previously described.^2^ ^20^ Sequential AFB procedures allow the collection of biopsies from both the same preinvasive lesion longitudinally and the detection and biopsy of new lesions either de novo or via spread from an initial lesion. Each lesion is biopsied by separate forceps to eliminate cross-contamination. Full informed consent was obtained from all patients.

### Tissue sectioning and laser capture microdissection

Approximately 10 serial sections of 8 μm thickness were cut from each formalin-fixed, paraffin embedded block. The first and last sections were stained with haematoxylin and eosin using a standard protocol and reviewed by two independent histopathologists to identify areas of histologically abnormal epithelium. Normal or preinvasive areas of the epithelium were then microdissected separately from methyl green-stained sections (Vector Laboratories, USA) using the PALM Microbeam system (Zeiss, Germany). Genomic DNA was extracted from the captured cells by digestion in PicoPure proteinase-K buffer (Arcturus, UK) according to the manufacturer's instructions. Tubes containing digestion buffer but no captured material were included in each DNA extraction batch and served as negative controls.

### Mutational detection

DNA from the invasive cancer or most recent CIS lesion was used to screen for somatic mutation(s) in *TP53* (exons 5–8), *K-RAS* (exon 1, codons 12–13) and *CDKN2A* (p16^INK4a^, exon 2) using nested-PCR. Preinvasive lesions collected longitudinally from each patient were then examined at those genomic loci mutated in the corresponding screening specimen. Constitutional DNA from whole blood was also examined in order to exclude the possibility of the detected genetic abnormality being a germline mutation. The obtained sequence electropherograms were compared with the published reference sequences using the basic local alignment search tool (BLAST). To determine the assay sensitivity and the percentage of *TP53* mutant cells that need to be present to allow detection of a mutation, we serially diluted tumour DNA carrying the *TP53* deletion with normal, wildtype control DNA. The sensitivity of the PCR-based detection method was determined to be ≤5%. PCR primer/product size details and amplification reactions are provided in the online data supplement (see online supplementary tables S1 and S2, respectively).

### Mutation verification

The Catalogue of Somatic Mutations in Cancer database (http://www.sanger.ac.uk/genetics/CGP/cosmic; Cambridge, UK) was used to verify all the somatic mutations identified. Nucleotide changes in the electropherogram were considered as true somatic mutations only when seen in the absence of any background noise/artefact and were accepted only if confirmed by repeating the PCR from the original DNA lysate.

### Loss of heterozygosity (*LoH*)

Constitutional blood DNA from patient 1 was amplified for three microsatellite markers (D17S 1506, D17S 1678 and D17S 1881) using Qiagen's Multiplex PCR kit (Crawley, UK) according to manufacturer's instructions and 5′-tagged primers with either HEX or FAM fluorescent probes. Preinvasive and control samples with sufficient DNA were subsequently analysed for *LoH* using only microsatellites found to be heterozygous in constitutional DNA. Fragment analysis was carried out on an ABI 3100, and data were analysed using PeakScanner V.1.0 (Applied Biosystems). *LoH* was calculated using the height ratio of the two-allele peaks in tissue DNA after normalisation to the allele peak heights from constitutional DNA. A change of at least twofold in the allelic ratio was considered as *LoH*. Microsatellite primer details for the *LoH* analysis are provided in online supplementary table S3.

## Results

Patients were selected from the UCLH Early Lung Cancer Detection Program. Of the 113 patients in this cohort with preinvasive lesions, 61 had follow-up data for more than 12 months, and 10 of these had an AFB detected and histologically confirmed high-grade (SD or CIS) lesion in the trachea. Eight of these had at least one visible high-grade lesion in the distal bronchial tree with seven having documentation of distal lesions prior to the tracheal CIS lesion. One of these patients had no biopsies available for analysis, and two had no mutations detected in the screened genes. The remaining five patients had longitudinally collected specimens with a somatic *TP53* mutation. Patient demographics, clinical presentation and circumstances leading to the detection of preinvasive lesions are summarised in table 1. All reported CIS lesions were detected by abnormal autofluorescence and histological confirmation. The number of months after initial presentation is documented as M_x_ (eg, month 12—M_12_). Full details for each patient's specimens are summarised in tables 2 and 3. Samples ranged from patient 1, who had 46 tissue samples analysed over a period of 12 years (2000–2012), to patient 4, who had three tissue samples analysed over 2 months.

**Table 1:** Demographic and clinical characteristics of the patients with tracheal carcinoma in situ included in the study

Patient	Sex	Smoking (pack years)	Initial malignancy (stage)*	Site	Age (surgery)	Site(s) of new tumour (histology/time to diagnosis†)	Sites tested	Specimens analysed
1	M	60	SqCC (T1N0M0)	LUL	58	Left completion pneumonectomy (SqCC) arising from a region of LUL stump (M_93_)	19	46
2	M	30–40	SqCC (T1N0M0)	LLL	72	LLL stump (SqCC/M_48_)	6	8
						Right Lung (SqCC/M_50_)		
						LUL (CIS /M_78_)		
						Trachea (SD /M_85_)		
3	M	47	SqCC (T1N0M0)	LUL	66	Lower Trachea/LMB stump (both early invasive SqCC/M_42_)	5	6
4	M	40	SqCC (T2N0M0)	RUL	57	RUL stump (SqCC/M_11_) Trachea posterior wall (invasive SqCC/M_11_)	3	3
5	M	40	SqCC (T1N0)	RML RLL	70	RIB (CIS /M_60_)	6	7

*Refers to initial malignancy detected prior to patient's referral to UCLH.

†Refers to the time interval between surgery and diagnosis of new SqCC/CIS.

CIS, carcinoma in situ; LLL, left lower lobe; LMB, left main bronchus; LUL, left upper lobe; RIB, right intermediate bronchus; RLL, right lower lobe; RML, right middle lobe; RUL, right upper lobe; SqCC, squamous cell carcinoma; SD, severe dysplasia; UCLH, University College London Hospital.

**Table 2:** Tracing the spread of the TP53 p.E294fs*51 (c.880delG) mutant clone over time in patient 1

AFB date	Month	Biopsy site	Diagnosis	AFB status	TP53 status	*LoH/RoH* (D17S 1881)
10/2000	M_38_	LUL stump	CIS	+	Mutant	LoH
		LLL orifice (control)	NAD	−	*Wildtype*	RoH
		LLL (control)	NAD	−	*Wildtype*	RoH
		*MC (control)	NAD	−	*Wildtype*	RoH/RoH
		RUL (control)	NAD	−	*Wildtype*	RoH
		RLL (control)	NAD	−	*Wildtype*	RoH
03/2001	M_43_	LUL stump	SD	+	Mutant	LoH
07/2001	M_47_	LUL stump	CIS	+	Mutant	LoH
12/2001	M_51_	LLL (control)	NAD	−	*Wildtype*	RoH
01/2003	M_64_	Left main bronchus	SD	+	Mutant	N/A
07/2003	M_70_	LLL (control)	NAD	−	*Wildtype*	RoH
		†LLL orifice	SD	+	Mutant	LoH /LoH
		Proximal left main bronchus	SD	+	Mutant	LoH
		Left main bronchus	CIS	+	Mutant	N/A
11/2003	M_74_	LLL (control)	NAD	−	*Wildtype*	N/A
		Proximal left main bronchus	CIS	+	Mutant	N/A
05/2004	M_80_	LLL (control)	NAD	−	*Wildtype*	N/A
09/2004	M_84_	LLL (control)	NAD	−	*Wildtype*	N/A
04/2006	M_104_	MC	CIS	+	Mutant	N/A
		MC (control)	NAD	−	*Wildtype*	N/A
08/2006	M_108_	Lower anterior trachea	CIS	+	Mutant	LoH
01/2007	M_113_	Lower lateral trachea	CIS	+	Mutant	LoH
04/2007	M_116_	Lower trachea	CIS	+	Mutant	N/A
03/2008	M_127_	RMB (control)	NAD	−	*Wildtype*	N/A
		RMB	MoD	+	Mutant	N/A
		Lower trachea (control)	NAD	−	*Wildtype*	*RoH*
08/2008	M_132_	MC/stump	CIS	+	Mutant	N/A
		Proximal RMB medial wall	CIS	+	Mutant	LoH
		Proximal RMB medial wall	SD	+	Mutant	N/A
01/2009	M_137_	RMB	CIS	+	Mutant	N/A
		LMB stump	CIS	+	Mutant	LoH
07/2009	M_143_	Lateral wall of orifice to RMB	CIS	+	Mutant	LoH
		Posterior wall proximal RMB	CIS	+	Mutant	LoH
		Left pneumonectomy stump	CIS	+	Mutant	LoH
11/2010	M_159_	RMB	MiD	−	*Wildtype*	RoH
		RMB	Metaplasia	−	*Wildtype*	RoH
		MC	CIS	+	Mutant	LoH
		MC	MiD	−	*Wildtype*	RoH
		MC (control)	NAD	−	*Wildtype*	RoH
		RML (control)	NAD	−	*Wildtype*	RoH
05/2011	M_165_	RML	CIS	+	Mutant	N/A
03/2012	M_175_	RML	CIS	+	Mutant	N/A
		RLL	CIS	+	Mutant	N/A
08/2012	M_180_	Posterior wall of RMB	SqCC	+	Mutant	N/A

Month refers to post-lobectomy.

*At M_38_, two distinct areas of the MC were biopsied, providing two AFB and histology normal samples.

†At M_70_, two distinct AFB detected and histology-confirmed SD samples from the LLL orifice were analysed and found to carry the c.880delG. In total, 46 samples from 19 spatially distinct areas were analysed over a period of 12 years (M_38_–M_180_).

AFB, auto-fluorescence bronchoscopy; CIS, carcinoma in situ; LLL, left lower lobe; LMB, left main bronchus; *LoH*, loss of heterozygosity; LUL, left upper lobe; MiD, mild dysplasia; MoD, moderate dysplasia; N/A, not analysed due to insufficient DNA lysate; NAD, no abnormality detected; RLL, right lower lobe; RMB, right main bronchus*;* RML, right middle lobe; *RoH*, retention of heterozygosity; RUL, right upper lobe; SD, severe dysplasia.

**Table 3:** Clonality assessment over time in patients 2, 3, 4 and 5

Patient	Bronchoscopy date	Time post-lobectomy (months)	Biopsy site	Diagnosis	Mutated gene (exon)	Mutation identified
2	07/2001	M_30_	MC (control)	NAD	*–*	*Wildtype*
	08/2002	M_43_	LUL	SD	*TP53-7*	p.C242F; c.725G>T
			LLL stump	SD	*TP53-7*	p.C242F; c.725G>T
	12/2002	M_47_	MC	MoD	*TP53-7*	p.C242F; c.725G>T
			MC	Metaplasia	*–*	*Wildtype*
			LUL/lingula sub	CIS	*TP53-7*	p.C242F; c.725G>T
	03/2003	M_50_	RLL segmentectomy	CA	*KRAS*	p.E88*; c.262G>T
	02/2006	M_85_	Trachea	CA	*TP53-7*	p.C242F; c.725G>T
3	05/2003	M_3_	MC (control)	NAD	*–*	*Wildtype*
	10/2003	M_8_	LUL stump	SD	*TP53-5*	p.V173L; c.517G>T
	08/2005	M_30_	Left pneumonectomy stump	SD/CIS		p.V173L; c.517G>T
	03/2006	M_37_	Trachea	CIS		p.V173L; c.517G>T
	08/2006	M_42_	Left pneumonectomy stump	SqCC		p.V173L; c.517G>T
			Lower trachea	SqCC		p.V173L; c.517G>T
4	07/2009	M_9_	Proximal RMB	CIS	*TP53-5*	p.V173L; c.517G>T p.V157F; c.469G>T
	09/2009	M_11_	RUL stump	SqCC	*TP53-5*	p.V173L; c.517G>T p.V157F; c.469G>T
			Posterior wall lower trachea	SqCC	*TP53-5*	p.V173L; c.517G>T
5	06/2004	M_50_	LLL (control)	NAD	*–*	*Wildtype*
			LUL (control)	NAD	*–*	*Wildtype*
			Right lobectomy stump	MoD/SD	*TP53-7*	p.C242R; c.724T>C p.G244D; c.731G>A
	11/2004	M_55_	Right lobectomy stump	SD		p.C242R; c.724T>C p.G244D; c.731G>A
	04/2005	M_60_	RIB	CIS/Inv.		p.C242R; c.724T>C p.G244D; c.731G>A
			Lower trachea	CIS		p.C242R; c.724T>C p.G244D; c.731G>A
	10/2005	M_66_	RMB polyp	SD		p.C242R; c.724T>C p.G244D; c.731G>A

CIS, carcinoma in situ; LLL, left lower lobe; LUL, left upper lobe; RIB, right intermediate bronchus; RLL, right lower lobe; RMB, right main bronchus; RUL, right upper lobe; SD, severe dysplasia.

### Clonally related preinvasive lesions appear over time and are spatially distinct

**Figure 1:**
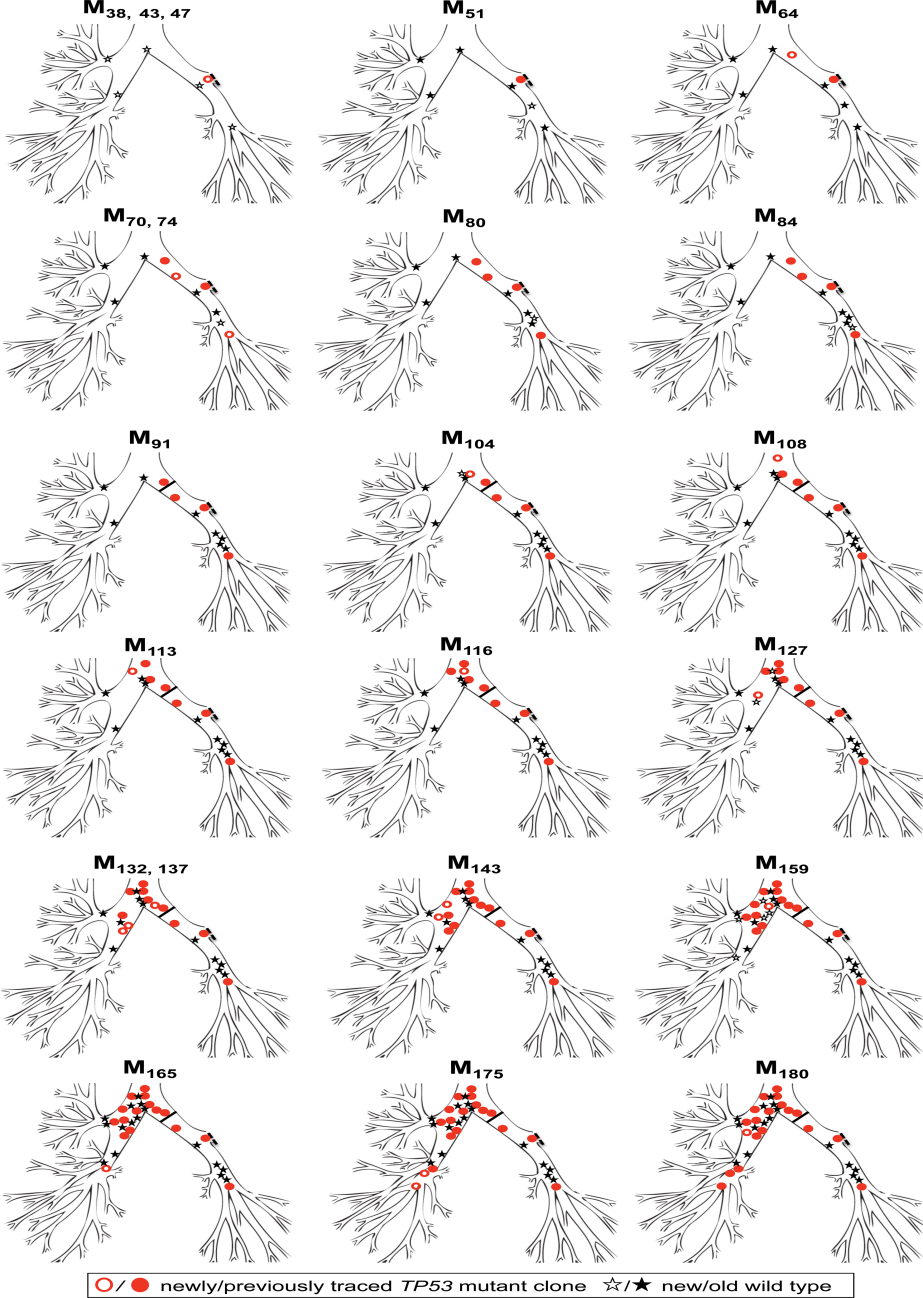
Spread of the *TP53* p.E294fs*51 mutant clone (exon 8) over time in patient 1. The mutant clone (red circle/filled red circle) was first detected at M_38_ in the left upper lobe (LUL) stump and spread over 142 months (M_38_–M_180_) in a distal-to-proximal direction towards the MC and trachea and then over into the right lung. Control biopsies from autofluorescence bronchoscopy-negative areas confirmed histologically normal and genetically wildtype intervening epithelium (shown as black star or filled black star). The part of the lung surgically removed by lobectomy (dashed black line, LUL stump) is not shown. Completion pneumonectomy (M_91_) is depicted as a solid black line.

Patient 1 underwent left upper lobectomy for SqCC (T1N0M0) in 1997 (tumour 1, M_0_). He had occasional episodes of haemoptysis but AFB was normal including the lobectomy stump (M_10_). At M_34_ after further haemoptysis, CIS was detected near the lobectomy stump and the patient entered the UCLH Early Lung Cancer Detection Program. Sequencing of this CIS lesion (M_38_) revealed the presence of a *TP53* p.E294fs*51 deletion (c.880delG, exon 8) (figure 1). At this time (M_38_), there were no further AFB-detectable lesions and normal epithelium (to AFB and histological analysis) biopsied as control tissue from six separate areas (figure 1) was *TP53* wildtype. The mutant clone remained at the resection margins at M_43_ and M_47_. At M_64_, a new AFB-detected spatially separate SD lesion in the left main bronchus (LMB) was positive for the *TP53* deletion. Three further spatially distinct lesions in the left bronchial tree were detected at M_70_ (both SD) and both harboured the identical mutant clone. Normal epithelium lay between each lesion (AFB-negative, histologically normal and *TP53* wildtype) confirmed at M_51_, M_70,_ M_74,_ M_80_ and M_84_. The *TP53* deletion remained in a subsequent biopsy from a resampled CIS lesion (M_74_). The patient underwent a completion left pneumonectomy (M_91_) after developing a further SqCC within the left lower lobe (LLL) (tumour 2), beyond the main airways detected by CT scan.

A new CIS lesion at the MC (M_104_), detected a year later, contained the same *TP53* deletion (after previously being normal and *TP53* wildtype at M_38_). Adjacent, control epithelium (AFB-negative, histologically normal) remained wildtype. Three subsequent bronchoscopies at M_108_, M_113_ and M_116_ detected new and spatially distinct CIS lesions in the lower trachea. All sites tested positive for the deletion, confirming a proximal spread of the mutant clone. At M_127_, the deletion was detected for the first time within the right main bronchus (RMB). Surrounding normal biopsies obtained at the same time were again wildtype. At M_132_, M_137_, M_143,_ M_165_ and M_175_, autofluorescence demonstrated further spatially distinct lesions of CIS and SD in the right lung and all contained the identical *TP53* deletion as detailed in table 2. Control biopsies from AFB normal sites showing normal or metaplastic areas of the right bronchial tree between AFB abnormal sites were *TP53* wildtype.

Importantly, a CIS specimen obtained at M_159_ from a resampled site in the MC was still found to carry the deletion, but normal and mild dysplastic epithelium laser captured from either side of the MC CIS lesion was again *TP53* wildtype. The *TP53* mutation was finally traced in an invasive SqCC specimen (tumour 3) from the posterior wall of the RMB at M_180_, almost 12 years after its first detection in the left upper lobe (LUL) stump (M_38_).

Based on these findings, we propose that the *TP53* p.E294fs*51 clone originated at the LUL either prior to or within the 38 months of the lobectomy and subsequently skipped towards the MC and trachea and then into the right lung, giving rise to spatially distinct and clonally related lesions (table 2, figure 1). DNA material from the original resected tumour (tumour 1, M_0_) was not available. Further evidence of their clonal relationship is provided by microsatellite–*LoH* analysis of a marker close to *TP53,* which showed a consistent loss of the same allele in all CIS/SD cases (table 2).

Of particular interest are the normal molecular findings of control biopsies taken in between lesions and the normal epithelium laser captured from the margins of lesions. Of the 46 biopsies analysed for *TP53*, 27 were CIS/SD (58.7%) and 19 were normal (41.3%). All CIS lesions were AFB-detected and found to harbour the *TP53* deletion. In contrast, all AFB-negative normal biopsies analysed were *TP53* wildtype, indicating a 100% correlation between AFB and mutational status.

After noting the distal-to-proximal spread of these lesions into the trachea, we went on to determine whether other patients with tracheal CIS lesions had the same chronology and clonal relationship.

### Tracheal CIS occurs after secondary spread from a distal clonally related preinvasive lesion

**Figure 2:**
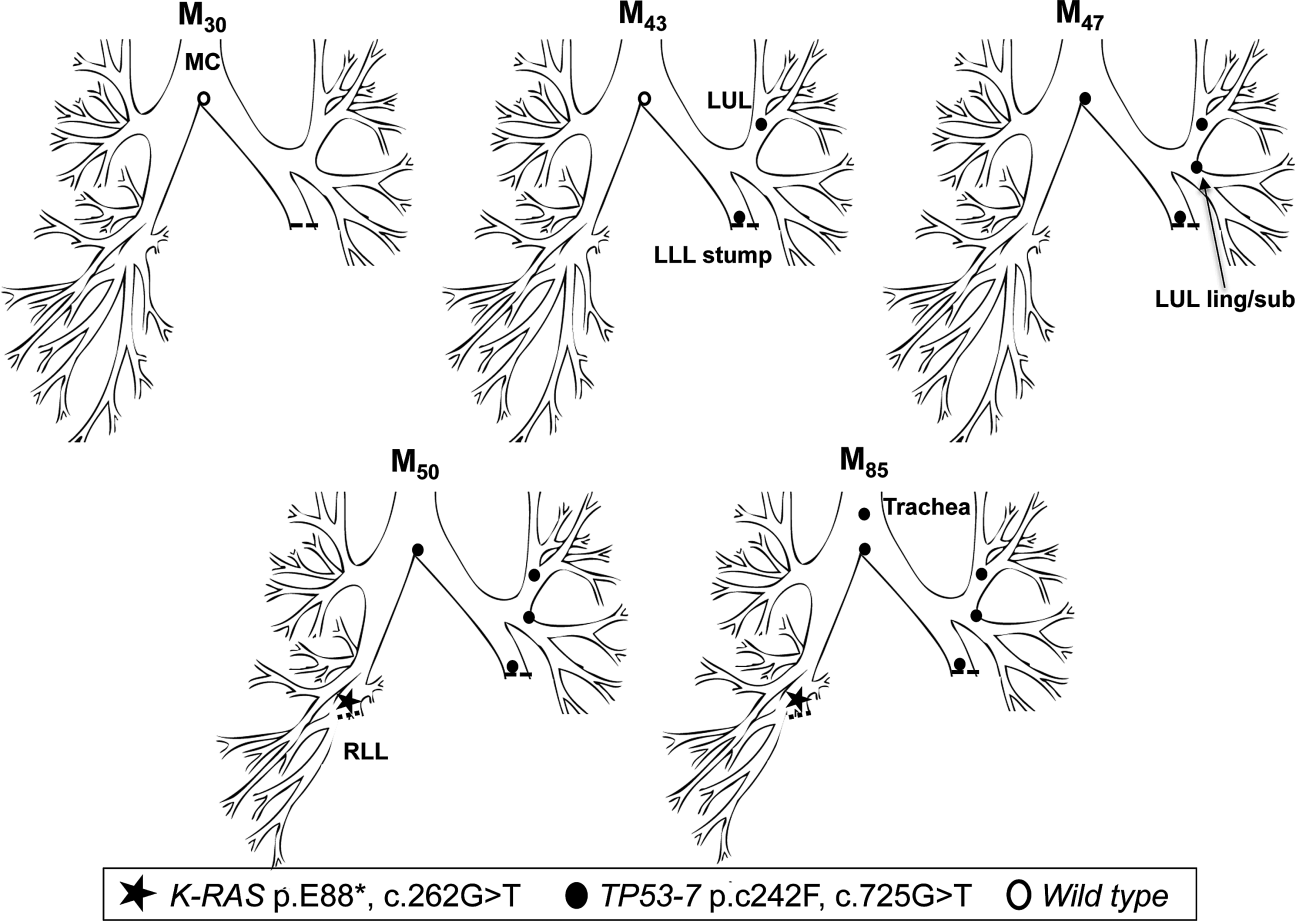
Tracing of the common mutant clone over time in the tracheobronchial tree of patient 2. Tracing the *TP53* c.725G>T clone (exon 7) over 42 months from the left lower lobe stump (M_43_) to the trachea (M_85_). A second, unrelated tumour (*K-RAS* c.262G>T) was detected in the right lower lobe at M_50_.

In early 1999 (M_0_), patient 2 underwent a left lower lobectomy for SqCC (tumour 1, T1N0M0). CIS at the LLL stump resection margin was detected, and he entered into the surveillance programme. Over the following 47 months, he developed three new preinvasive lesions, each containing an identical *TP53* p.C242F, c.725G>T mutation (exon 7) (figure 2). The mutant clone spread proximally from the LLL to LUL/lingula and towards the MC. At M_50_, a CT scan and biopsy revealed a T1N0M0 poorly differentiated SqCC beyond the bronchoscopically visible airway in the right lung. Molecular analysis of the resected cancer revealed the presence of a *K-RAS* p.E88*, c.262G>T mutation but no *TP53* mutation, indicating that this was an unrelated cancer (tumour 2). The CIS lesions within the airway were static by AFB for three and a half years, but the *TP53* mutant clone was subsequently detected in a new lesion at M_85_ in the trachea (figure 2). Histology showed an area of CIS with local invasion (tumour 3; table 3).

**Figure 3:**
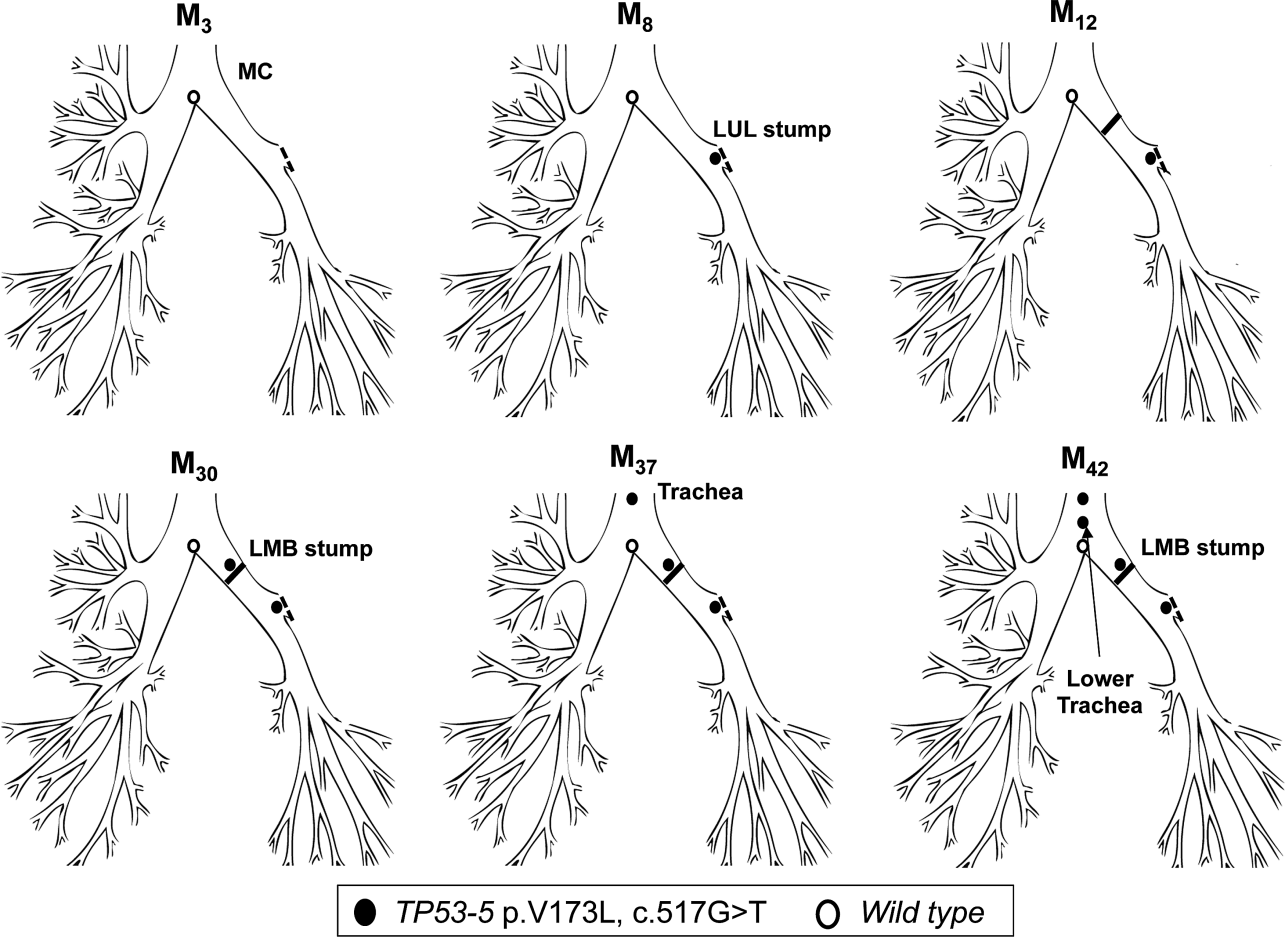
Tracing of the common mutant clone over time in the tracheobronchial tree of patient 3. Distal-to-proximal spread of the c.517G>T clone (*TP53* exon 5) within 29 months from the left upper lobe stump (M_8_) to the trachea (M_37_). The clone must have arisen at the lower trachea earlier than M_37_.

Patient 3 had a left upper lobectomy for SqCC (T1N0M0) in February 2003 (M_0_) and has since been under surveillance for CIS at the resection margin. A *TP53* p.V173L, c.517G>T mutation was detected in a resection margin specimen at M_8,_ while a normal sample taken from the main carina at M_3_ was wildtype. At M_12_, the patient underwent a left completion pneumonectomy for clinical suspicion of invasion due to nodularity of the stump CIS lesion, but no evidence of microinvasive disease was found at pathology. Continued AFB surveillance later found the same mutation at the pneumonectomy stump (M_30_), and at an AFB distinct lesion in the trachea (M_37_). The patient eventually developed two microinvasive carcinomas at the pneumonectomy stump and trachea (M_42_), both positive for the c.517G>T mutation (figure 3; table 3), for which he received photodynamic therapy at M_44_. We have shown previously the lesion at M_8_ and the pneumonectomy stump carcinoma at M_42_ to be monoclonal in origin based on a shared common 3q telomeric amplicon.^21^

**Figure 4:**
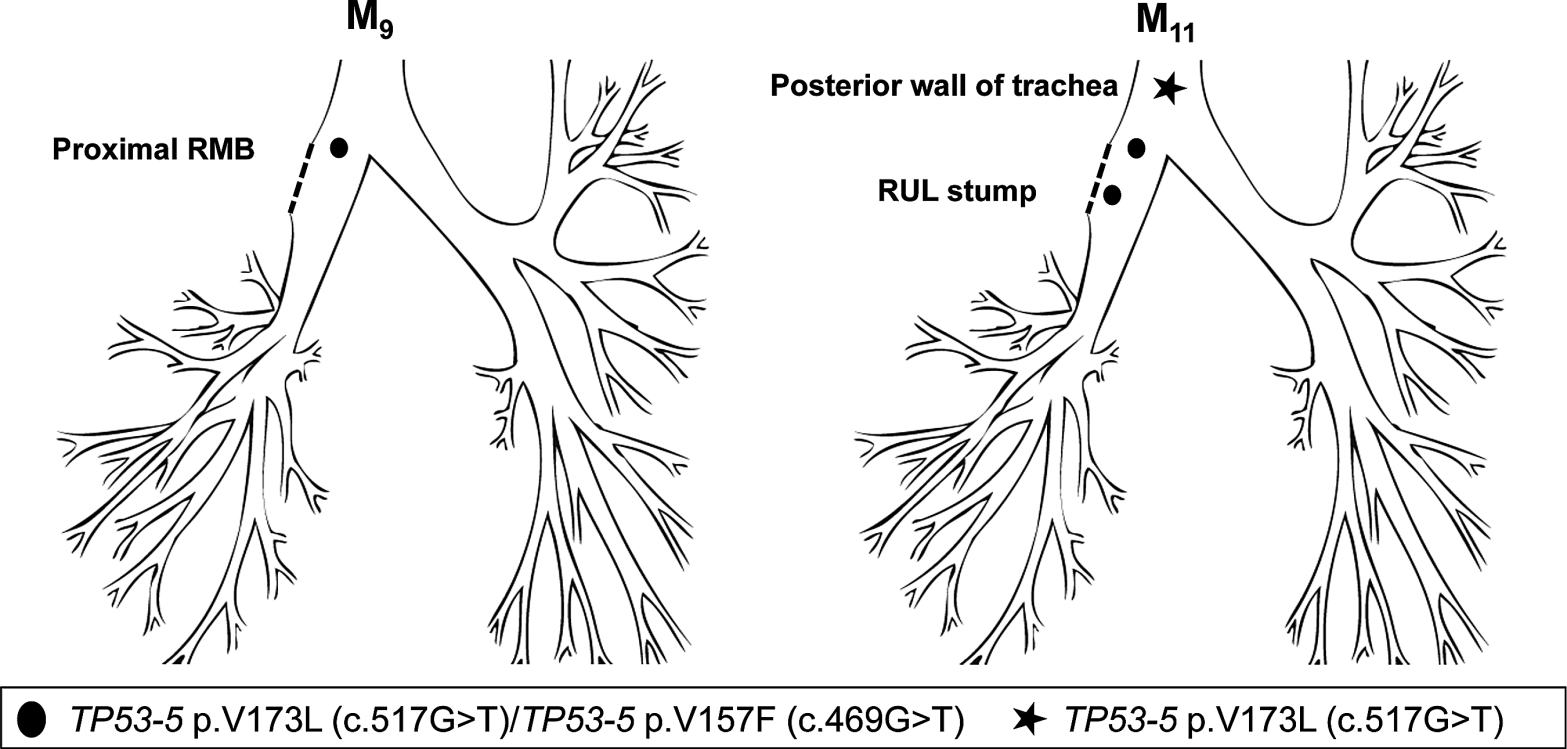
Tracing of the common mutant clone over time in the tracheobronchial tree of patient 4. Detection of the c.517G>T and c.469G>T mutations (both *TP53* exon 5) at the right upper lobe stump (M_11_). Only one mutation was detected at the posterior wall of the trachea (M_11_), suggesting this was the first mutation and these cells spread towards this site before the second mutation occurred.

Patient 4 underwent a right upper lobectomy in October 2008 (M_0_) for poorly differentiated SqCC (T2N0). CIS at the resection margin was histologically confirmed, and the patient was referred for surveillance. A sample of this lesion showed two *TP53* mutations: c.517G>T (p.V173L) and c.469G>T (p.V157F) that matched a later developing CIS lesion obtained from the proximal RMB. The resection margin lesion progressed to invasive cancer at M_11_. Interestingly a second SqCC, detected at M_11_ within the posterior wall of the lower trachea, was found to harbour the c.517G>T clone but not the c.469G>T, indicating the c.517G>T was the earlier mutation and cell migration from the original lesion occurred before the second mutation (figure 4; table 3).

**Figure 5:**
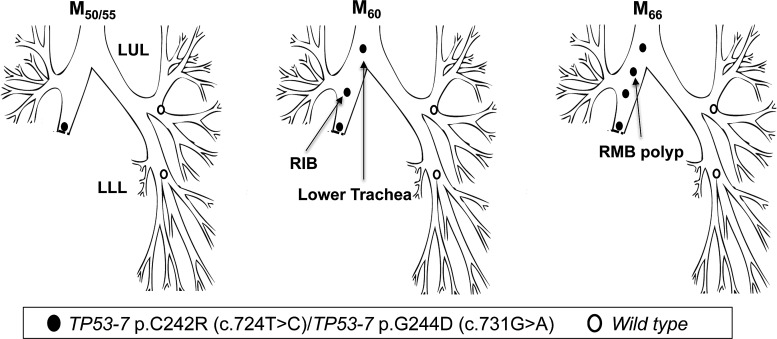
Tracing of the common mutant clone over time in the tracheobronchial tree of patient 5. Simultaneous detection of the c.724T>C and c.731G>A clones (both *TP53* exon 7) at the lobectomy stump 50 months postsurgery (M_50_) and distal-to-proximal spread towards the lower trachea.

Patient 5 underwent a right lower and middle lobectomy for SqCC (T1N0) in April 2000 (M_0_). Residual CIS was reported at the resection margin. At referral to the centre (M_50_), AFB detected MoD/SD at the lobectomy stump with genetic analysis revealing two *TP53* mutations: p.C242R, c.724T>C and p.G244D; c.731G>A (both in exon 7), with neither of the two mutant clones present in normal epithelium from the LUL and LLL. At M_60_, the same mutations were detected in two new lesions, one more proximally in the right intermediate bronchus (RIB, CIS with microinvasion) and a second in the lower trachea (CIS) (figure 5; table 3). At M_66_, a further lesion was detected in the RMB (SD) again harbouring both mutations.

Hence, all five patients developed clonally related but spatially distinct lesions over time spreading from distal-to-proximal airways.

## Discussion

We have used genetic analysis to define the natural history and clonal spread of preinvasive lesions identified by repeat AFB of the tracheobronchial tree in patients over periods of up to 12 years. In doing so, we have been able to document in situ the generation of multiple preinvasive lung cancer lesions giving new insight into field cancerisation of the lungs. Further, we show in all five patients with tracheal CIS that disease starts in the bronchial tree and moves proximally upwards into the trachea forming spatially distinct lesions.

The data presented here confirm that multifocal preinvasive lesions most frequently derive from a common clonal ancestor as suggested by the seminal paper by Franklin and colleagues^15^ rather than arising independently.^14^ Intriguingly, our AFB and genetic data show these lesions, while clonal are not contiguous, suggesting that cellular migration has occurred through the bronchial tree, with subsequent clonal expansion in a new environment. One remarkable patient demonstrates clonally related but physically distinct preinvasive lesions that moved in a distal-to-proximal spread from the left bronchial tree, up into the trachea, and down into the right bronchial tree over a period of 12 years. This raises significant biological questions regarding the mobility of airway epithelium and the evolutionary dynamics of the mutant clones. The distance travelled by spatially distinct clonal lesions over time gives us some idea of the rate of mutant cell migration. If we examine patient 1, the mutant clone moves from the LUL entrance to the right lower lobe. This is 10.8 cm, indicating a migration rate of 0.075 cm/month.

**Figure 6:**
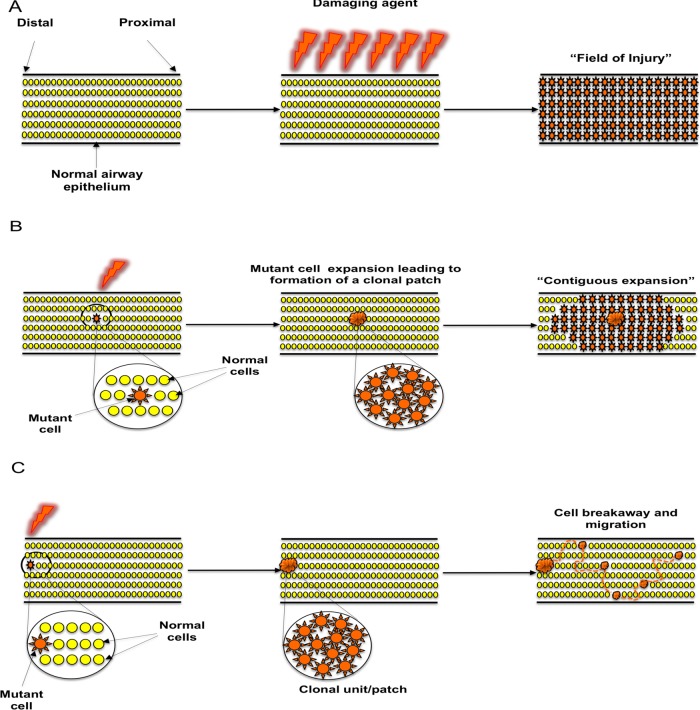
Proposed mechanisms of clonal spread of carcinoma in situ in the lung epithelium. (A) ‘Field of injury’. Growth of clonally unrelated, multifocal lesions within a field predisposed to the same change after exposure to a common damaging agent. (B) ‘Contiguous field’. Continuous expansion of a clone, giving rise to a patch of clonally related cells and formation of a progressively expanding contiguous field. (C) ‘Cell breakaway and migration’. Following formation of a ‘clonal unit’ from an initial cell that acquired a proliferative advantage through acquisition of a mutation, a cell or group of cells breaks away from the unit and migrates via histologically normal and genetically wildtype epithelium in order to establish new lesions within a suitable niche.

Our results support the concept of ‘field cancerisation’ in the lung but not by a mechanism previously postulated. Previous hypotheses include the ‘field of injury’ hypothesis (figure 6A), where all cells in a defined area are exposed to a damaging agent delivering the same insult to every cell.^22^ Alternatively, an individual cell clone has been postulated to expand relentlessly, possibly in response to injury, setting up a contiguous field of a mutant clone in which invasive disease can develop (figure 6B).^23–25^ Our results clearly mitigate against the latter hypothesis by demonstrating neighbouring, intervening epithelium between clonally related preinvasive lesions to be both normal in histology and wildtype for the genetic loci tested.

It is possible that we have missed thin snakes of CIS, migrating through the normal epithelium joining the lesions; however, we feel this is unlikely for a number of reasons. First, our PCR-detection method detects mutant *TP53* at levels below 5% when mixed with wildtype DNA. Furthermore, the longitudinal nature of our sampling would be expected to detect intervening abnormal cells over time. The LLL of patient 1 was tested wildtype at six different time points (M_38_–M_84;_ table 2). Even if very few mutant CIS cells were initially present at the LLL at M_38_ that went undetected due to sensitivity issues, it might be expected to be able to find the mutation at a later time point after cell expansion. There has also never been any autofluorescent evidence of a snake of cells in any of the patients screened within our surveillance study.

The hypothesis that these lesions occur independently would mean that in our first patient the identical *TP53* deletion occurred on at least 19 occasions, leading to pathologically identical but clonally separate lesions. The detected *TP53* deletion does not lie within the mutational *TP53* hotspot sites,^26^ and the statistical chances of the identical single deletion occurring in each of the samples sequenced are infinitesimally small. Our patient 1 data clearly show clonal expansion. The c.880delG mutation is reported just seven times in the total of 3128 simple *TP53* sequence variants reported in the COSMIC mutation database.^27^ Assuming the mutation frequency of approximately 0.002 is representative of all neoplasia, the chance of two *TP53* mutant cells both having the same c.880delG mutation is less than 5×10^−6^. This is further supported by the observation that all preinvasive lesions also had the same pattern of allelic loss on 17p, implying that the c.880delG mutation was always present on the retained allele. Taken together, these findings suggest that it is highly unlikely for two unrelated cells to independently develop the same pattern of genetic defects, strongly indicating that these cells share a recent common clonal ancestor.

Our data therefore indicate subsequent preinvasive lesions occur as a result of individual cells or clusters of cells migrating and becoming established in a new environment where they experience a selective growth advantage (figure 6C). Interestingly, once on the bronchoscopy surveillance programme, all five patients quit smoking, indicating that the developments documented here are not related to new smoking damage. A final explanation for the apparent discontinuity of mutant cells could be ‘healing’ of the wildtype epithelium and concomitant fragmentation of the mutant cells, but this does not fit with the AFB findings.

These data are interesting when considering the details of clonal evolution in the preneoplastic lung. The extensive clonal expansion of *TP53* mutant cells implies selection for these mutants, perhaps a consequence of dysregulated apoptotic and senescence machinery. However, the ‘patchiness’ of mutants suggests that the fitness may be slight, temporal or related to environmental factors, such that genetically wildtype cells are able to intervene between, and so presumably outcompete, the mutant cells. Our data suggest that the evolutionary dynamics constrain the clonal expansion of *TP53* mutants such that they are unable to repopulate the entire epithelium in a contiguous sheet.

We cannot determine in our studies the cell of origin of these lesions, although by their nature the lesions consist of keratin 14-expressing basal cells recently proposed to be progenitor cells of the upper airway.^28^ ^29^ Long lifespan, self-renewal and the ability to replicate for extended periods of time support the hypothesis of stem or progenitor cell-induced tumourigenesis.

Our study provides further evidence that longitudinally occurring preinvasive lesions are clonally related and shows for the first time that these lesions are actually spatially distinct. These data catalogue field cancerisation in the lung and suggest that the evolutionary dynamics restrict mutant clones to grow in spatially distinct lesions rather than repopulating the entire epithelium. In addition, we document for the first time that tracheal CIS frequently develops from a distal CIS lesion in the bronchial tree. Our findings, therefore, demonstrate that while individual premalignant lesions may appear stable, they are capable of intra-airway migration. Currently it is not possible to predict which lesions will spread or progress to invasive cancers. Further study of the lesions that progress to cancer compared with those that remain stable or regress may provide molecular targets for the design of novel therapeutic approaches. Currently there are no randomised studies describing the best approach of treating these lesions. Treatments, however, should be tissue sparing because further lesions are likely to occur. Importantly, post-treatment, surveillance with detailed autofluorescent examination over extended time periods must also be offered to patients in order to detect subsequent developing CIS lesions that had metastasised pretreatment.

## Supplementary Material

Web supplement
