# Supplementary material for: Cell migration leads to spatially distinct but clonally related airway cancer precursors
Source: Thorax. 2014 Feb 18;69(6):548–57. doi: 10.1136/thoraxjnl-2013-204198 (PMC4033139; doi:10.1136/thoraxjnl-2013-204198)
Supplement: Web supplement [file thoraxjnl-2013-204198-s1.pdf]

Supplements

Cell migration leads to spatially distinct but clonally related airway cancer precursors

Christodoulos P Pipinikas[1], Theodoros S Kiropoulos[1, 2], Vitor H Teixeira[1], James M Brown[1], Aikaterini Varanou[1], Mary Falzon[3], Arrigo Capitanio[3], Steven E Bottoms[1], Bernadette Carroll[4], Neal Navani[1, 4], Frank McCaughan[5, 6], Jeremy P George[4], Adam Giangreco[1], Nicholas A Wright[7, 8], Stuart A C McDonald[7, 8], Trevor A Graham[7, 9], Sam M Janes[1, *]

Table S1. Primer Details

Gene	Exon	[A] Primer	First Round	Product size (bp)	Second Round	Product size (bp)
TP53	5	F	CACTTGTGCCCTGACTTTCA	307	TCTGTCTCCTTCCTCTTCCTACA	245
		R	GAGCAATCAGTGAGGAATCAGA		AACCAGCCCTGTCGTCTCT	
	6	F	AGAGACGACAGGGCTGGTT	237	CAGGCCTCTGATTCCTCACT	182
		R	TGGAGGGCCACTGACAAC		CTTAACCCCTCCTCCCAGAG	
	7	F	TGCTTGCCACAGGTCTCC	236	CTTGGGCCTGTGTTATCTCC	161
		R	GGTCAGAGGCAAGCAGAGG		GTGTGCAGGGTGGCAAGT	
	8	F	TTTTTAAATGGGACAGGTAGGA	258	GCCTCTTGCTTCTCTTTTCC	191
		R	CACCCTTGGTCTCCTCCAC		GCTTCTTGTCCTGCTTGCTT	
CDKN2A (p16)	2A	F	GCTTCCTTTCCGTCATGC	300	CCTGGCTCTGACCATTCTGT	245
		R	CAGGTACCGTGCGACATC		CAGCTCCTCAGCCAGGTC	
	2B	F	CTGTTCTCTCTGGCAGGTCA	394	CTTCCTGGACACGCTGGT	234
		R	TGTGCTGGAAAATGAATGCT		TGGAAGCTCTCAGGGTACAAA	
K-RAS	–	F	GAGTTTGTATTAAAAGGTACTGGTGGA	272	TTTGATAGTGTATTAACCTTAT	210
		R	ATCAAAGAATGGTCCTGCAC		TATTAAAACAAGATTTACCTC	

[A] Dried desalted primers were from Sigma-Aldrich (Dorset, UK)

Table S2. PCR Amplification Reactions

	TP53-5	*TP53-6*	*TP53-7*	*TP53-8*	*p16-2A*	*p16-2B*	*K-RAS*
**First Round PCR**							
	**[A]1/5/55**	**2/5/60**	**1/5/60**	**2/5/60**	**2/5/60**	**2/5/60**	**2/5/60**
**Template**	2.00	2.00	2.00	2.00	2.00	2.00	2.00
**[B] Buffer**	2.50	2.50	2.50	2.50	2.50	2.50	2.50
**[C] $MgCl_2$**	1.00	2.00	1.00	2.00	2.00	2.00	2.00
**[D] dNTP Mix**	0.50	0.50	0.50	0.50	0.50	0.50	0.50
**Q-solution**	5.00	5.00	5.00	5.00	5.00	5.00	5.00
**[E] Forward primer**	0.25	0.25	0.25	0.25	0.25	0.25	0.25
**Reverse primer**	0.25	0.25	0.25	0.25	0.25	0.25	0.25
**Taq**	0.20	0.20	0.20	0.20	0.20	0.20	0.20
**H_20**	13.30	12.30	13.30	12.30	12.30	12.30	12.30
**Second Round PCR**							
	**1/5/60**	**1/0/60**	**1/5/60**	**2/0/60**	**2/5/60**	**2/5/60**	**2/5/55**
**Template**	2.00	2.00	2.00	2.00	2.00	2.00	2.00
**Buffer**	2.50	2.50	2.50	2.50	2.50	2.50	2.50
**$MgCl_2$**	1.00	1.00	1.00	2.00	2.00	2.00	2.00
**dNTP Mix (10mM)**	0.50	0.50	0.50	0.50	0.50	0.50	0.50
**Q-solution**	5.00	0.00	5.00	0.00	5.00	5.00	5.00
**Forward primer**	0.25	0.25	0.25	0.25	0.25	0.25	0.25
**Reverse primer**	0.25	0.25	0.25	0.25	0.25	0.25	0.25
**Taq**	0.20	0.20	0.20	0.20	0.20	0.20	0.20
**H_20**	13.30	18.30	13.30	17.30	12.30	12.30	12.30

[A] Numbers refer to volume of $MgCl_2$ and Q-solution in the reaction and PCR annealing temperature (°C), respectively
[B] Promega 10x reaction buffer without $MgCl_2$ used at the supplied concentration (Southampton, UK)
[C] $MgCl_2$, Q-solution and Taq DNA polymerase are all from Qiagen (Crawley, UK)
[D] dNTP mix working concentration was 10mM (Invitrogen, Paisley, UK)
[E] Primer working concentration was 20μM

Table S3. Microsatellite primer details for multiplex *LoH* analysis

[A] Marker	Location	Sequence (5'-3')	Expected product size (bp)	Reaction Conditions
D17S 1678 (F)	17p	[6FAM] TTTGGGTCTTTGAACCCTTG	120	Multiplex PCR Mix: 25µl<br>[B] 10x Primer Mix (2µM each primer): 5µl<br>RNase Free-H_2O: 18µl<br>Template DNA: 2µl<br><br>Annealing Temperature: 57°C
D17S 1678 (R)		CCACAACAAAACACCAGTGC		
D17S 1506 (F)	17q	[HEX] TGTGGGATGGGGTGAGATTTC	150	
D17S 1506 (R)		CTGTTGGTCGGTGGGTTG		
D17S 1881 (F)	17p	[6FAM] CCCAGTTTAAGGAGTTTGGC	210	
D17S 1881 (R)		TAGGGCAGTCAGCCTTGTG		

[A] Primers were HPLC purified and were reconstituted in TE buffer at a final stock concentration of 100µM.

[B] For each microsatellite marker, 10µl of the 100µM stock were mixed together and the volume was adjusted to 500µl using distilled H_2O. The final concentration of each primer in the working primer mix was 2µM. The final concentration of each primer used in the amplification reaction was 0.2µM.
